# Harnessing the potential of therapeutic agents to safeguard bone health in prostate cancer

**DOI:** 10.1038/s41391-018-0060-y

**Published:** 2018-07-09

**Authors:** Kurt Miller, Günther G. Steger, Daniela Niepel, Diana Lüftner

**Affiliations:** 1 Department of Urology, Charité–Universitätsmedizin Berlin, Berlin, Germany; 2 Department of Medicine, Clinical Division of Oncology, Comprehensive Cancer Centre, Medical University of Vienna, Vienna, Austria; 3 Amgen Europe GmbH, Zug, Switzerland; 4 Department of Hematology and Oncology, Charité–Universitätsmedizin Berlin, Berlin, Germany

## Abstract

**Background:**

Patients with prostate cancer are at risk of impaired bone health. Prostate cancer has a propensity to metastasize to bone, after which patients are at risk of skeletal-related events (SREs). These complications are associated with increased mortality, substantial pain, and reduced quality of life. Patients are also at risk of bone loss due to androgen deprivation therapy (ADT), which can be compounded in elderly patients with reduced bone density. It is essential, therefore, that aspects of bone health and therapies able to prevent the occurrence of SREs are considered throughout the clinical course of prostate cancer.

**Methods:**

We reviewed the literature regarding the molecular mechanisms underpinning bone lesion formation, the modes of action of therapies that prevent SREs, and the efficacy and safety of these therapies in patients with hormone-sensitive or castration-resistant prostate cancer (CRPC).

**Results:**

Therapies such as denosumab (a RANKL inhibitor) and zoledronic acid (a bisphosphonate) were indicated for prevention of SREs. Radium-223 dichloride also has proven efficacy in delaying symptomatic SREs, as well as in improving overall survival through effects on bone metastases. Before development of bone metastases, low-dose denosumab may also be used for treatment of ADT-associated bone loss. Denosumab may also have the potential to delay bone metastases development in patients with CRPC, although this is not currently an approved indication. The safety profile of therapies to prevent SREs should be considered. This review consolidates the available evidence on use of denosumab and bisphosphonates in prostate cancer, differentiated by hormone-sensitive and castration-resistant disease.

**Conclusions:**

There is convincing evidence to support the use of denosumab and bisphosphonates to maintain bone health in patients with prostate cancer. Clinicians should be mindful of the adverse event risk profile of these therapies.

## Introduction

The skeleton is a common site of metastases in prostate cancer; indeed, more than 90% of patients with metastatic, castration-resistant prostate cancer (mCRPC) have evidence of bone metastases [1, 2]. The factors that determine the location of secondary tumors are complex; however, blood flow patterns and cell signaling pathways, such as the C-X-C motif chemokine 12–C-X-C chemokine receptor type 4 axis, are both influential [3, 4]. The propensity for bone metastases also reflects the favorable microenvironment that results from release of growth factors during bone resorption [5–7].

Patients with bone metastases may develop skeletal complications, known as skeletal-related events (SREs), which include pathologic fracture, radiation, or surgery to bone and spinal cord compression [8]. Spinal cord compression is of particular concern in prostate cancer given the high frequency of metastases at this site [2, 9] and its debilitating consequences, which can include paralysis [8, 9]. However, pathologic fracture and radiation to bone (used to treat bone pain) are also common in this population owing to bone instability in osteoblastic metastatic lesions [7, 10, 11]. Patients with bone metastases can experience multiple SREs each year [12], placing a considerable burden on patients and healthcare systems [11, 13]. SREs are associated with increased mortality, substantial pain, and reduced quality of life (QoL) [13–17].

Further complicating the preservation of bone health in prostate cancer is the fact that androgen deprivation therapy (ADT) causes considerable reductions in serum testosterone and estradiol levels, leading to cancer treatment-induced bone loss (CTIBL) and an increased fracture risk proportional to the treatment duration [18, 19]. It is important to bear in mind that patients with prostate cancer are typically elderly, with impaired bone strength before ADT is initiated. For example, a study of 348 men (median age 55.4 years) found that prevalence of osteoporosis in those aged over 50 years was ~19%, and, in a study of 618 men with newly diagnosed advanced prostate cancer starting ADT treatment (mean patient age 73 years), 80% had abnormal bone mineral density (BMD) at baseline [20, 21].

The maintenance of bone health is therefore central to all stages of prostate cancer treatment. The management of patients with bone metastases focuses on preventing SREs, palliating pain, and maintaining QoL [22]. The inhibitors of bone resorption, zoledronic acid (a bisphosphonate) and denosumab (a receptor activator of nuclear factor-kappaB [23] ligand [RANKL] inhibitor), are approved for SRE prevention in patients with solid tumors metastatic to bone [24, 25]. In addition, the bone-seeking radiopharmaceutical radium-223 dichloride (radium-223) has been approved for treatment of castration-resistant prostate cancer (CRPC) with symptomatic bone metastases [26]. Denosumab is also approved for protecting against ADT-induced bone loss (at a lower dose than that indicated for bone metastases) [25]. Denosumab and bisphosphonates have been investigated in other roles, such as in bone metastases prevention [27, 28]. Understanding the role that denosumab and bisphosphonates may have at various stages is imperative to ensure that patients with prostate cancer receive optimal care.

This review summarizes the molecular mechanisms underpinning bone lesion formation, together with the modes of action through which bisphosphonates and denosumab prevent SREs. Bone health in patients with hormone-sensitive prostate cancer and in those with castrate-resistant disease is discussed. Safety considerations when using agents to prevent SREs are surveyed, and an overview is given of ways in which use of bisphosphonates or denosumab can be optimized in current clinical practice.

### Molecular basis of CTIBL and bone metastasis

In healthy adults, bone is in a perpetual state of remodeling, a process that is essential to preserve structural integrity and minimize the risk of fragility fractures [29]. Bone remodeling involves several cell types, including osteoclasts (cells that resorb bone), osteoblasts (cells that produce and secrete osteocalcin and calcified matrix) and osteocytes (cells that regulate osteoclast development); bone homeostasis relies on precise signaling between the three cell types [3].

Reductions in estrogen levels, induced by ADT, lead to dysregulation of bone remodeling through the parathyroid-mediated activation of osteoclasts [30, 31]. Circulating tumor cells that settle in bone also alter the delicate signaling balance between osteoblasts, osteoclasts, and osteocytes [3]. Tumor cells release a range of factors that stimulate osteoclast activity or alter osteoblast function. This can result in an increase in bone resorption (leading to osteolytic lesions) or an increase in irregular formation of poor-quality bone (causing osteoblastic lesions); [32, 33] in the case of prostate cancer, the latter predominates. Both osteolytic and osteoblastic lesions increase the risk of fracture and other SREs compared with that observed in healthy bone [34].

Molecules that are formed during absorption or formation of bone are used as biomarkers to identify and assess the extent of bone metastases in patients with solid tumors [35–37]. Examples of established bone turnover biomarkers include N- and C-terminal cross-linked telopeptides of type I collagen (both markers of bone breakdown), procollagen type I N-terminal peptides and bone-alkaline phosphatase (BALP; both markers of bone formation) [29, 36, 38]. A raised BALP level can indicate bone metastasis and is associated with a poor prognosis [36]. Accordingly, it has been recommended that plasma BALP should be measured in baseline assessments of prostate cancer [39–41].

### Mechanisms of action of bisphosphonates and denosumab

Nitrogen-containing bisphosphonates are small molecules that dock in hydroxyapatite binding sites on bone surfaces. When osteoclasts begin to resorb bisphosphonate-impregnated bone, bisphosphonates are liberated and bind to farnesyl pyrophosphate synthase inside the osteoclasts, ultimately leading to apoptosis [24, 42, 43]. Denosumab is a fully humanized monoclonal antibody, which has a different mechanism of action to bisphosphonates; it targets and binds to RANKL, preventing activation of RANK on the surface of osteoclasts. Inhibition of the RANKL–RANK interaction impedes osteoclast formation, function, and survival, thereby decreasing bone resorption [34].

### Bone health in patients with hormone-sensitive prostate cancer

In early-stage prostate cancer, ADT is the mainstay of therapy. Treatment guidelines, such as those from the European Society for Medical Oncology (ESMO), recommend ADT (e.g., gonadotropin-releasing hormone [GnRH] agonists or antagonists) alongside surgical and radiotherapy options in the management of locally advanced prostate cancer, as well as high-risk localized prostate cancer [44]. ADT is also recommended for metastatic, hormone-sensitive prostate cancer (mHSPC) [44].

ADT causes CTIBL in patients with prostate cancer [45]. Reductions in BMD of 5–10% are typical in the first year after ADT initiation [18]. This translates into an increased fracture risk that is proportional to ADT duration [19]. In a 7-year cohort study using data from a United States (US) claims database, clinical fractures in men with GnRH-agonist-treated, non-metastatic prostate cancer increased by 21% relative to matched, untreated patients [46]. Although some additional fractures may have been related to bone metastases, a longer treatment duration conferred a greater fracture risk [46]. In a New Zealand population-based cohort study, ADT was associated with a significantly increased risk of any fracture and hip fracture requiring hospitalization. Data from Canada, China, and the United Kingdom (UK) have also demonstrated an increased fracture risk following ADT [47–49].

**Fig. 1:**
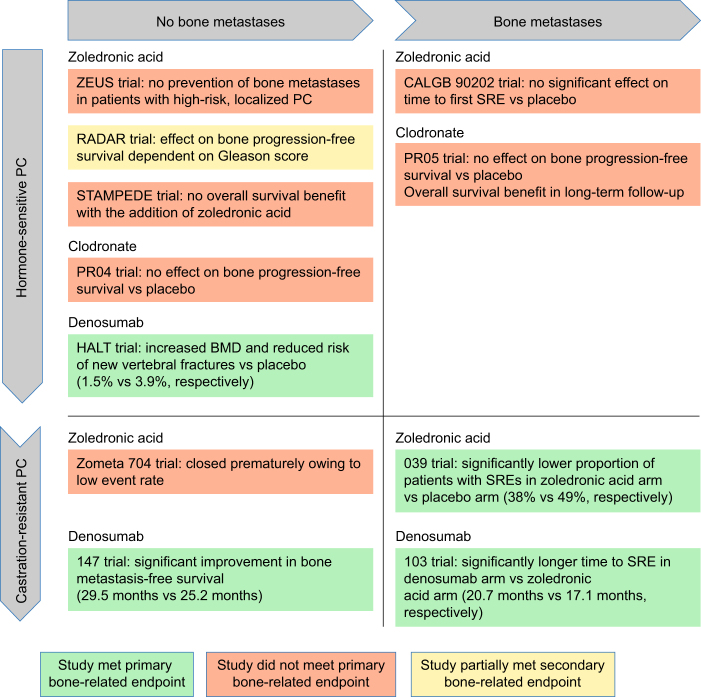
Summary of phase 3 trials investigating the use of antiresorptive drugs in the control of hormone-sensitive and CRPC with and without bone metastases

Denosumab and bisphosphonates may also promote bone health in non-metastatic HSPC. Significant improvements in BMD have been reported in patients receiving additional zoledronic acid, alendronate or risedronate vs placebo [50–52], and zoledronic acid and pamidronate both prevented BMD loss in ADT-treated patients (Fig. 1) [53, 54]. However, none of the bisphosphonates are approved for CTIBL treatment [29]. In contrast, the RANKL inhibitor denosumab is indicated for prevention of ADT-associated bone loss in men with increased fracture risk [55]. In a placebo-controlled study of denosumab given at the licensed dose of 60 mg subcutaneously (SC) every 6 months to 1468 men receiving ADT for non-metastatic HSPC, 36 months of denosumab treatment led to increased BMD compared with baseline at all sites and a reduction in the incidence of new vertebral fractures (1.5% with denosumab vs 3.9% with placebo; relative risk: 0.38; 95% confidence interval [CI]: 0.19–0.78; *p* = 0.006) [56]. Beneficial effects of denosumab over placebo were also observed in patient subgroups defined by age, duration and type of previous ADT, BMD *T*-score, weight, body mass index, bone turnover marker levels, and number of prevalent vertebral fractures [57].

Both high-dose denosumab and high-dose zoledronic acid, are indicated for prevention of SREs in adults with bone metastases from prostate cancer [24, 25]. Although these indications include mHSPC, regulatory trials for both agents were conducted in mCRPC, and effects in HSPC have not been fully investigated. Early use of intravenous (IV) zoledronic acid 4 mg every 4 weeks, as an ADT adjunct to prevent SREs in mHSPC, was evaluated in the phase 3 Cancer and Leukaemia Group B (CALGB) 90202 study; however, it was terminated early because no beneficial effect on time to first SRE was observed with zoledronic acid [58]. Nonetheless, it has been suggested that further data analysis for patients with decreased baseline BMD could help to evaluate whether some subgroups may benefit from zoledronic acid in the hormone-sensitive stage [59].

Another aspect to be considered at the hormone-sensitive stage is the potential for therapeutic intervention to delay disease progression. The Androgen Blockade Therapy With or Without Zoledronic Acid in Treating Patients With Prostate Cancer and Bone Metastases (ZAPCA) trial in men with treatment-naive, metastatic prostate cancer indicated that zoledronic acid and ADT may delay development of castration resistance in a subset with low baseline, prostate-specific antigen values [60]. Data from a preclinical study in hypogonadal mice inoculated with human prostate cancer cell lines suggested that androgen depletion accelerated bone metastasis. Of note, bone metastasis reduced when the mice received zoledronic acid [32]. Based on these observations, denosumab and bisphosphonates might have been expected to have a positive survival effect in HSPC. However, the controlled, multiple-arm Systemic Therapy in Advancing or Metastatic Prostate Cancer: Evaluation of Drug Efficacy (STAMPEDE) study showed that, for 2962 patients with HSPC with and without metastases receiving standard of care (ADT plus optional radiotherapy), addition of zoledronic acid (4 mg every 3–4 weeks) and docetaxel, or zoledronic acid alone, showed no improvement in survival compared with docetaxel and standard of care [61]. Similarly, addition of zoledronic acid and celecoxib to standard of care also failed to improve survival significantly, although it did improve survival in a pre-planned subgroup analysis of men with metastatic cancer [62]. The lack of survival impact of zoledronic acid added to standard of care was corroborated by a meta-analysis of studies in HSPC [63]. Findings from the Medical Research Council (MRC) PR04 and PR05 studies of clodronate and ADT showed some overall survival benefit in patients with metastases compared with placebo (hazard ratio [HR]: 0.77; 95% CI: 0.60–0.98; *p* = 0.032), but not in those without metastases (HR: 1.12; 95% CI: 0.89–1.42; *p* = 0.94) [64]. The PR05 study showed no significant bone progression-free survival benefit in patients with metastases (Fig. 1) [65]. This lack of benefit could suggest that other mechanisms contribute to bone metastases development. For example, research suggests that human epidermal growth factor receptor 2 (HER2) and epidermal growth factor receptor (EGFR) are upregulated in response to ADT; [66, 67] in addition, a recent study demonstrated that EGFR promotes the survival of prostatic tumor infiltrating cells and circulating tumor cells that metastasize to bone, and that HER2 supports the growth of prostate cancer cells once they are established at the site of metastasis [68]. Levels of RANK and RANKL expression have been shown to be higher in aggressive metastatic prostate cancer cells than that in cells removed from primary tumors, lending support to a hypothesis that osteoclast mediated bone resorption my stimulate colonization and progression of metastases in bone [69, 70]. It is possible that treatment regimens may need to target multiple mechanisms to increase survival effectively.

### Bone health in patients with castrate-resistant prostate cancer

Patients with HSPC might expect to progress to CRPC ~2–3 years after ADT initiation [71]; however, most patients with recurrent or metastatic HSPC who are treated with ADT progress more quickly (median 18–24 months) [72, 73]. SREs are associated with increased mortality in CRPC and bone metastases [74], and bone-related parameters are good overall survival prognostic variables [75]. Both zoledronic acid (4 mg IV every 3–4 weeks) and denosumab (120 mg SC every 4 weeks) are indicated for SRE prevention in patients with CRPC and existing bone metastases [12, 29, 76, 77]. In a placebo-controlled study of 643 men with CRPC and bone metastases, zoledronic acid treatment led to fewer SREs (38% vs 49% with placebo; *p* = 0.028) and reduced the overall skeletal complications risk by 36% [12]. Treatment with denosumab has shown superiority to zoledronic acid for time to first SRE, and to second and subsequent SREs, in a phase 3, placebo-controlled, double-blind study of 1904 men with CRPC and bone metastases [77]. Time to first SRE was extended from 17.1 to 20.7 months (HR: 0.82; 95% CI: 0.71–0.95; *p* = 0.008 for superiority). Second and subsequent SREs were also delayed, resulting in an 18% reduction in cumulative SREs [77]. In addition, a post hoc analysis has shown that denosumab reduced the risk of symptomatic skeletal events (SSEs) compared with zoledronic acid [78].

Skeletal pain is a complication of bone metastases that has a substantial impact on QoL [17] and may respond poorly to treatment. In addition to direct effects on bone health, denosumab and bisphosphonate treatment can ameliorate bone pain [13, 79]. In a pooled analysis of data from three phase 3 studies in patients with bone metastases from CRPC, breast cancer and other solid tumors, denosumab was superior to zoledronic acid with regard to pain improvement and interference [13, 79]. Denosumab treatment delayed the onset of moderate/severe pain by 1.8 months (median: 6.5 vs 4.7 months; HR: 0.83; 95% CI: 0.76–0.92; *p* < 0.001) and clinically meaningful increases in overall pain interference by 2.6 months (median: 10.3 vs 7.7 months; HR: 0.83; 95% CI: 0.75–0.92; *p* < 0.001) compared with zoledronic acid. Fewer denosumab-treated patients required strong opioids or reported worsening of clinically meaningful health-related QoL compared with patients treated with zoledronic acid [79].

In addition to denosumab and zoledronic acid, a different class of agent has emerged. Radium-223 is a calcium mimetic that binds preferentially to newly formed bone in metastases; [80] its benefits in mCRPC were demonstrated in the phase 3 Alpharadin in Symptomatic Prostate Cancer Patients (ALSYMPCA) trial, in which it was compared with placebo when added to standard of care.1 The trial was terminated early after a planned interim analysis demonstrated a significant overall survival benefit of radium-223, which was the primary endpoint.1 Updated analyses demonstrated continued survival improvement (14.9 vs 11.3 months with radium-223 vs placebo, respectively; HR: 0.70; 95% CI: 0.58–0.83; *p* < 0.001).1 Radium-223 also significantly prolonged time to first SSE compared with placebo (median: 15.6 vs 9.8 months; HR: 0.66; 95% CI: 0.52–0.83; *p* < 0.001).1 The survival benefits of radium-223 have been corroborated by results of a study of patients with mCRPC and symptomatic or asymptomatic bone metastases enrolled in an early access program, in which median overall survival was 16 months [81]. Significant pain reduction (*p* = 0.035) was demonstrated within 2 weeks in a phase 2 trial, with a dose-dependent pain response observed in up to 71% of patients after 8 weeks [82].

It has been suggested that denosumab and bisphosphonates can prevent development of bone metastases in patients with CRPC; however, this has not been demonstrated consistently. Zoledronic acid therapy, for example, has failed to result in any clear bone metastases prevention in both HSPC and CRPC studies [28]. In the Zometa European Study (ZEUS) of zoledronic acid (4 mg every 3 months) in 1433 men with high risk, localized prostate cancer, no significant improvement in bone metastasis-free survival was observed after a median follow-up of 4.8 years (Fig. 1) [28]. However, in a phase 3, double-blind, randomized, placebo-controlled study of men with non-metastatic CRPC at high risk of bone metastases, denosumab (120 mg every 4 weeks) significantly increased bone metastasis-free survival (by a median of 4.2 months vs placebo; HR: 0.85; 95% CI: 0.73–0.98; *p* = 0.028) and delayed time to first bone metastases (33.2 vs 29.5 months with placebo; HR: 0.84; 95% CI: 0.71–0.98; *p* = 0.032). Overall survival did not differ between the groups [27]. Nonetheless, denosumab is not indicated in patients without bone metastases, possibly because the difference in bone metastasis-free survival was not deemed to be clinically meaningful by regulators [83]. Further studies may be warranted to determine effects of denosumab and bisphosphonate on prevention of bone metastases in CRPC; this is particularly relevant for patients with short prostate-specific antigen (PSA) doubling times, as a study has demonstrated that PSA velocity is an independent predictor of time to first bone metastasis [27, 28, 59, 84]. In addition, more evidence is required to determine whether delaying bone metastases can reduce SREs. This information will help to inform the benefit–risk profile of long-term treatment with denosumab and bisphosphonates.

### Key safety considerations for denosumab and bisphosphonates

Denosumab and bisphosphonate safety profiles have been described previously and are generally acceptable [12, 76, 77, 85]. However, adverse events should be considered when assessing the benefit–risk ratio, particularly given the likelihood that denosumab and bisphosphonates will be used for longer in clinical practice than has been investigated historically [59].

Denosumab and bisphosphonates inhibit osteoclastic bone resorption and reduce skeletal calcium release; consequently, they are associated with a hypocalcemia risk [86]. In the comparative study of denosumab 120 mg and zoledronic acid 4 mg every 4 weeks, hypocalcemia occurred in 13% and 6% of patients, respectively [77]. The higher hypocalcemia incidence with denosumab vs zoledronic acid was consistent with its greater antiresorptive effect [25, 77, 86]. Hypocalcemia appears to occur most often during the initial stages of therapy, but stabilizes thereafter and does not increase with exposure duration [86]. Label guidelines for zoledronic acid and denosumab advise calcium and vitamin D supplementation, pre-treatment low serum vitamin D and calcium correction, and serum calcium monitoring during treatment [24, 25, 55].

Denosumab and bisphosphonates have also been associated with an elevated osteonecrosis of the jaw (ONJ) risk, although it was not recognized as an adverse event of interest at the time of the zoledronic acid initial studies [76]. A small proportion of patients with prostate cancer reported this adverse event in the comparative study (denosumab, 2.3%; zoledronic acid, 1.3%; *p* = 0.09) [77]. The incidence of ONJ increases with time, and is more common in patients with poor oral health or a tooth extraction history [87]. Recent safety findings from the comparative study open-label extension showed an ONJ incidence (unadjusted for exposure) of 8.2% for patients with metastatic prostate cancer who received denosumab (120 mg every 4 weeks); cumulative ONJ incidence was 3.8% when given for up to 5.6 years [85]. For patients who initially received zoledronic acid (4 mg every 4 weeks) and then switched to denosumab, ONJ incidence was 5.9% over 5 years; cumulative incidence was 2.2% over a period of up to 3.4 years [85]. In the overall study analysis (patients with breast or prostate cancers), patient-year adjusted incidence of confirmed ONJ was 1.1% during the first treatment year, 3.7% during the second and 4.6% per year thereafter [88]. To reduce ONJ risk, ESMO guidelines recommend preventative dental measures before starting treatment, and good oral hygiene maintenance and invasive dental procedure avoidance during therapy [29].

Zoledronic acid is not metabolized and is excreted unchanged via the kidneys. It is not indicated for patients with severe renal impairment (creatinine clearance < 30 mL/min); also, it should be used with caution in mild-to-moderate renal impairment and may require dose adjustment [24]. For denosumab, renal monitoring and dose adjustments are not needed, including in severe renal impairment [25, 89]. However, individuals with severe renal impairment are at risk of hypocalcemia and should be monitored closely [25].

Radiopharmaceutical agents have historically been associated with a myelosuppression risk [90]. Radium-223, however, offers highly localized tissue destruction with low levels of myelosuppression [80]. The ALSYMPCA trial provided evidence that radium-223 might be better tolerated than older generation radiopharmaceuticals; incidences of grades 3 and 4 anemia, neutropenia and thrombocytopenia were minimal (13%, 3.1%, and 6.1%, respectively) [1].

### Current practice with denosumab and bisphosphonates in prostate cancer

Clearly, proactive management of bone health is important in prostate cancer. In clinical practice, however, many patients do not undergo bone density assessment. For instance, in a Canadian survey of 156 prostate cancer specialists, only 32.5% reported routinely measuring BMD before starting ADT and 36.6% did so 1–2 years after treatment initiation [91]. This is probably a barrier to appropriate prescription of denosumab and bisphosphonates in prostate cancer management.

There is some suggestion that denosumab and bisphosphonate prescribing behavior may vary according to the prescriber’s specialty. An analysis of denosumab and bisphosphonate real-world treatment patterns across six European Union countries was conducted using data from 1971 patients with prostate cancer and bone metastases [92]. This revealed that patients were more likely to receive denosumab and bisphosphonates if treated by an oncologist rather than a urologist (78% vs 60%, respectively), and treatment was more likely to be initiated early (56% vs 43%, respectively) [92]. Low rates of bisphosphonate treatment, and long delays between diagnosing bone metastases and initiating therapy, have also been reported in US community-based urology group practices [93].

### Optimizing the use of denosumab and bisphosphonates in clinical practice

Evidence suggests that denosumab and bisphosphonates may promote bone health in patients with prostate cancer. However, integration of these agents into disease management is constrained by their licensed indications and is complicated by the evolving treatment landscape. As with any clinical intervention, it is important to evaluate the benefit–risk profile when considering the role of these agents. For example, while the risk of hypocalcemia peaks early, ONJ incidence increases with treatment duration [86, 87]. This safety profile must be considered when initiating denosumab and bisphosphonate therapy, in order not to restrict their availability later on when patients are at a higher risk of SREs. The point at which denosumab and bisphosphonates therapy should be initiated, and their doses subsequently increased, remains unclear. A lack of data exists that compares high-dose denosumab and bisphosphonate efficacy for SRE prevention in HSPC, and also in early- vs late-stage mCRPC; such data would help to determine the point at which treatment should be started.

Such uncertainties regarding initiation and dosing of these agents are common to all cancers associated with bone metastases; therefore, ESMO has produced guidelines on bone health in patients with cancer [29]. These guidelines recommend that denosumab or zoledronic acid therapy ‘should be’ commenced on diagnosing bone metastases, and ‘should’ continue indefinitely and throughout the disease course [29]; however, evidence suggests this may not happen in clinical practice [94]. Denosumab and bisphosphonates have a role in treating CTIBL in men receiving ADT [57, 95, 96], and their use is ‘generally recommended’ by the ESMO guidelines [29], although only denosumab is licensed for this indication [55]. The ESMO guidelines also recognize that denosumab has been shown to delay bone metastases in CRPC [27, 29], and ‘generally recommend’ its use [29], although denosumab is not licensed for this indication [25]. No recommendations relating to use of radium-223 feature in this guidance [29].

In addition to the general ESMO guidelines on bone health in cancer [29], recommendations for use of denosumab and bisphosphonates in patients with prostate cancer are available in a number of clinical practice guidelines [29, 39, 40, 44, 97]. However, recommendations across these guidelines are inconsistent, which may suggest a need for more data to inform when to use denosumab and bisphosphonates in mCRPC. For instance, the ESMO prostate cancer guidelines recommend denosumab or zoledronic acid for patients with bone metastases from CRPC at high risk of clinically significant SREs [44], which is inconsistent with the aforementioned general ESMO bone health in cancer guideline recommendation for use of these agents in all patients with bone metastases [29]. The ESMO prostate cancer guidelines do not make recommendations regarding optimal treatment duration and dosing. They advise that men receiving long-term ADT should be monitored for side effects, including osteoporosis, although they do not make recommendations regarding use of denosumab or bisphosphonates in patients receiving ADT [44]. Radium-223 is supported as a first- or second-line mCRPC treatment option [44].

The St Gallen Advanced Prostate Cancer Consensus Conference guidelines recommend that the majority of men with CRPC and bone metastases should receive an osteoclast-targeted agent for SRE and SSE prevention [39]. However, the optimal time to initiate treatment, and treatment intensity and duration are unclear in these guidelines [39]. Radium-223 is not supported for routine use as a first-line treatment in patients with mCRPC [39].

The European Association of Urology prostate cancer guidelines recommend that denosumab or zoledronic acid may be offered to men with CRPC and skeletal metastases, along with calcium and vitamin D supplementation, to prevent osseous complications [40]. These guidelines also state that, for patients receiving ADT, preventative treatment with low-dose bisphosphonates or denosumab could be considered in individuals at risk of fracture. A dual-energy, X-ray absorptiometry scan is recommended for patients initiating ADT; bisphosphonates or denosumab should be considered for those with a *T*-score of less than –2.5 [40]. They also highlight the survival benefit reported for radium-223 in CRPC with symptomatic bone metastases, in those patients ineligible for, or whose disease is progressing after, docetaxel treatment [40].

An International Society of Geriatric Oncology (SIOG) position paper has highlighted the need to focus on bone health among elderly men with prostate cancer, owing in part to the steady decline in BMD that occurs with aging [97]. The paper indicates that, despite the value of agents such as denosumab and bisphosphonates in elderly patients with cancer, they are typically underutilized. The SIOG recommends that men aged over [75] years treated with ADT for prostate cancer should receive denosumab and bisphosphonates at doses used to prevent osteoporosis, and supports treatment initiation as soon as bone metastases are diagnosed to delay SREs and reduce complications. With regard to safety, consideration is needed of age-associated increases in hypocalcemia, vitamin D deficiency and dental disease risk [97]. Treatment choice may also be guided by an elderly patient’s renal function, with denosumab requiring no dose adjustment in cases of renal impairment [25].

The Cancer Care Ontario guidelines state that use of denosumab at a dose of [60] mg every 6 months should be considered to reduce fracture risk in men receiving ADT with non-metastatic prostate cancer at high risk of fracture. Denosumab at a dose of 120 mg every 4 weeks is recommended to prevent or delay SREs in patients with non-symptomatic mCRPC [98]. Bisphosphonates, at doses indicated for metastatic bone disease, are recommended for the same purpose in those with mildly symptomatic or asymptomatic CRPC. No medication is recommended to prevent development of bone metastases in patients with non-metastatic prostate cancer [98]. The guidelines suggest that radium-223 should be considered for the reduction of SSEs, to improve health-related QoL and to extend overall survival in patients with symptomatic mCRPC [98].

The benefits of denosumab and bisphosphonates in patients with prostate cancer become increasingly apparent as the disease progresses to CRPC and metastasizes to bone [59]. The role of these agents in CRPC is influenced, however, by the impact of other treatments on bone health. New-generation hormonal agents (abiraterone and enzalutamide) are now available and their influence on bone health must be considered. Both abiraterone and enzalutamide have been shown to prevent SREs and delay pain progression in patients with mCRPC [99, 100], although it has not been established whether these benefits are mediated by the antineoplastic effects of these agents or whether there is also an effect on the interaction between tumor cells and bone [59]. Data have also suggested that adding abiraterone to ADT may also delay SSEs in mHSPC [101]. Few data are available regarding denosumab and bisphosphonate effects when used in combination with these new-generation hormonal agents. However, a post hoc analysis of data from both treatment arms of the COU-AA-302 trial of abiraterone in mCRPC has suggested improved overall survival in patients receiving concomitant denosumab, zoledronic acid or other similar agents [102]. Data were also reported from a phase 3 study of 839 patients from an early access program suggesting survival benefits from combining radium-223 with abiraterone or enzalutamide, or both, or with denosumab [81].

There are no high-quality data to guide treatment duration with high-dose denosumab or bisphosphonates in patients with bone metastases, and expert opinion is divided on this topic; [39] however, reducing the dose frequency after an initial high-dose period is practiced and has been incorporated in clinical practice guidelines. For example, the ESO (European School of Oncology)–ESMO advanced breast cancer consensus guidelines suggest that it may be a reasonable compromise to replace 4-weekly with 3-monthly IV zoledronic acid therapy after an initial 1-year period [103]. Evidence in support of this practice in prostate cancer is emerging; a recent study has suggested the non-inferiority of 12- vs 4-weekly zoledronic acid regimens with respect to preventing SREs in patients with prostate cancer metastatic to bone (and also breast cancer and multiple myeloma) [104]. A similar trial evaluating denosumab scheduling with respect to preventing SSEs is ongoing (NCT02051218) [105].

Although there are apparent inconsistencies regarding denosumab and bisphosphonate initiation and dosing across guideline recommendations, the licensed indications of denosumab (120 mg every 4 week) and zoledronic acid (4 mg every 3–4 weeks) for prevention of SREs in adults with bone metastases from solid tumors [25], or advanced malignancies involving bone [24], respectively, do not specify the need for a SRE risk assessment or symptomatic bone metastases. These indications are informed by phase 3 trial data in which patients with current or previous objective evidence of bone metastases were treated without reference to their SRE risk [12, 27].

In addition to the benefits that might be achieved with denosumab and bisphosphonate use in prostate cancer, it is important to consider the implications of maintaining bone health on cost and healthcare resource utilization (HRU). Few studies directly examine the cost and HRU of CTIBL in prostate cancer, but it is well established that fractures secondary to osteoporosis, such as those caused by CTIBL, can result in severe pain, fatigue, functional impairment and a mortality of up to 20%, and can incur a significant cost to healthcare systems [106]. Evidence from the prospective, multinational, observational STARS study [107], and a European illness cost study [13], has illustrated the substantial cost and burden on HRU associated with SREs.

## Conclusions

Denosumab and zoledronic acid, as well as radium-223, have an established role in SRE prevention in mCRPC. Evidence suggests that denosumab and bisphosphonates can also protect bone health earlier in the course of prostate cancer. Recommendations for best practice on using these agents in patients with prostate cancer are available from a number of guidelines. However, it is apparent that there is a lack of consensus—based on a paucity of supporting data—relating to the practical integration of denosumab and bisphosphonates into prostate cancer management algorithms. As for other cancers, clarification is needed with regard to the time at which denosumab and bisphosphonates should be initiated in early-stage prostate cancer, and how long patients should be treated with low-dose denosumab and bisphosphonates before switching to high-dose. More data are required to determine the relative levels of early treatment benefit and risk, which may then be reflected in product licenses. With investigations ongoing, recommendations for the optimal integration of these agents in prostate cancer management should become clearer. When considered overall, there is convincing evidence to support use of denosumab and bisphosphonates to maintain bone health in patients with cancer, provided that clinicians are also mindful of the adverse events risk profile of these agents.
